# Adding value to mechanical ventilation

**DOI:** 10.1590/S1806-37132014000500002

**Published:** 2014

**Authors:** Guilherme Schettino

**Affiliations:** Department of Critical Care Medicine, Hospital Israelita Albert Einstein, São Paulo, Brazil

Value can be defined as a cost-to-result ratio. Better results, better delivery of certain processes, or lower costs translate to higher value. This concept has currently been used with propriety in health care as well, reinforcing the idea that it is necessary to obtain the best possible results, such as survival, functional independence, and satisfaction, at the lowest possible cost. This takes on added importance when we remember that health care resources, whether funds, personnel, or equipment, are finite, and doing more with less is crucial to provide care to all who need. Let us then imagine the value, for a mechanically ventilated patient, of adequate analgesia, of a protective ventilatory strategy, of a weaning protocol, of elevation of the head of the bed, of personnel hand-washing-all of which are interventions that produce excellent results and are quite inexpensive. The Brazilian recommendations for mechanical ventilation, the main objective of which is to add value to mechanical ventilation, were published in two parts, because of their length and scope, by two Brazilian journals: the Brazilian Journal of Pulmonology^(^ 1 ^,^ 2 ^)^ and the Brazilian Journal of Intensive Care Medicine.^(^ 3 ^,^ 4 ^)^

Respiratory failure is a common disease, and, in potentially reversible cases, ventilatory support is life-saving. Extrapolating epidemiological data from the United States^(^ 5 ^)^-2.8 mechanically ventilated patients per 1,000 population/year-to Brazil (current population of 203,175,000 inhabitants, according to the Brazilian Institute of Geography and Statistics),^(^ 6 ^)^ we can estimate that approximately 570,000 patients require invasive ventilatory support every year. Assuming a mean duration of mechanical ventilation of three days, we reach a figure of 1,706,670 ventilator days, and, on the basis of information from the 2011 Census by the Brazilian Association of Intensive Care Medicine,^(^ 7 ^)^ which calculated that there are approximately 25,000 ICU beds in Brazil, we can estimate that, every day, 19% of the ICU beds are used by intubated patients. In addition, the aforementioned data from the United States,^(^ 5 ^)^ according to which the cost of hospitalization for patients with respiratory failure and requiring mechanical ventilation is estimated to be US$34,000, allow us to extrapolate that Brazil will spend nearly R$54.5 billion/year, considering 12% of healthcare expenditures^(^ 8 ^)^ and 1.1% of the gross domestic product,^9^^)^ on hospital treatment of patients with acute respiratory failure or acute exacerbation of chronic respiratory failure.

The figures presented above, bearing in mind that they are the result of a simple epidemiological, mathematical, and financial exercise, draw attention to the huge impact that respiratory failure and mechanical ventilation have on heath policy in Brazil. However, it is important to remember that ventilatory support is known to be a cost-effective treatment for most patients. Studies published in recent years have shown figures ranging from US$26,000 to US$175,000 per quality-adjusted life year (QALY), depending on the etiology of respiratory failure, comorbidities, and patient age.^(^ 10 ^)^ Although arbitrary, it is current practice to accept treatments resulting in US$50,000-150,000/QALY as cost-effective.^(^ 11 ^)^

The published recommendations^(^ 1 ^-^ 4 ^)^ state that the results of treatment of patients with acute respiratory failure have improved greatly in recent decades, and what is most interesting is that this advance is more attributable to a better understanding of the pathophysiology of respiratory failure and to the prevention of ventilator-associated complications than to the development of new drugs or technologies. Mechanical ventilators, in their basic essence, have changed very little over this period, but the way they are used has changed completely, evolving from an aggressive strategy to correct hypoxemia and/or hypercapnia to a strategy focused on delivering a minimum alveolar ventilation to ensure gas exchange, sparing the lungs from further injury and thereby providing the time needed for lung recovery. Brazil has played a decisive role in the development of these modern concepts of mechanical ventilation, particularly in the understanding of the pathophysiology of ARDS and in being a pioneer in demonstrating the benefits of using protective ventilatory strategies. ^(^ 12 ^,^ 13 ^)^ Nevertheless, recent data have shown that the mortality rate of mechanically ventilated patients in Brazil remains high when compared with those found in developed countries.^(^ 14 ^)^ Limited access to ICU beds, the unsatisfactory number and poor training of health professionals assigned to the care of patients with respiratory failure, obsolete equipment, and, especially, the lack of adherence to best care practices are factors that certainly contribute to this worrisome finding.

The 2013 Brazilian recommendations for mechanical ventilation^(^ 1 ^-^ 4 ^)^ are an important initiative. Having been made by competent and experienced professionals, they represent the state of the art in mechanical ventilation, presented in a clear and objective manner and with a view toward adjustment to the way critical care medicine is practiced in Brazil.

Despite acknowledging all of the individual and collective efforts by the authors and coordinators of that work, we need to be aware that this is the easiest step in the quest for better care for mechanically ventilated patients; the hard part, the great challenge, not only here but worldwide, is to transform recommendations and good intentions into value for patients.^(^ 15 ^)^ I emphasize that most of those recommendations do not require new technologies or greater financial resources and are, for the most part, intuitive and already known by professionals working in ICUs in Brazil. We have another complicating factor: how to implement them in a country such as Brazil-a heterogeneous country of continental dimensions, where we are creative but have little discipline to follow recommendations, there is a lack of qualified professionals, there is no culture of training and continuing professional education for health professionals, and there is little measurement of the actual quality of care delivered by public or private health care institutions.

The authors of those recommendations^(^ 1 ^-^ 4 ^)^ have done their part, and we have another excellent guide toward delivering higher-quality, safer, and higher-value mechanical ventilation to patients with respiratory failure. The content and the rationale are laid out; now it is time to transform the evidence and recommendations into practice, and this will only happen with work, discipline, and involvement from each of us. Let us get to work!
