# Supplementary material for: Adding value to mechanical ventilation
Source: J Bras Pneumol. 2014 Sep-Oct;40(5):455–7. doi: 10.1590/S1806-37132014000500002 (PMC4263327; doi:10.1590/S1806-37132014000500002)
Supplement: Translation

# Translation: Agregando valor à ventilação mecânica

**Authors:** Guilherme Schettino

**Affiliations:** Departamento de Pacientes Graves, Hospital Israelita Albert Einstein, São Paulo (SP) Brasil

Valor pode ser definido como a relação entre resultado e custo. Quanto melhor o resultado, melhor a entrega de determinado processo ou menor o seu custo, maior será o valor. Esse conceito tem sido utilizado atualmente, com propriedade, também para a saúde, reforçando a ideia de que é preciso obter os melhores resultados possíveis, como, por exemplo, sobrevida, independência funcional e satisfação, com os menores custos. Isso ganha importância ainda maior quando lembramos que os recursos, sejam eles monetários, humanos ou tecnológicos para a saúde, são finitos, e fazer mais com menos é decisivo para disponibilizar cuidado a todos que precisam. Imaginemos, então, o valor, para um paciente em ventilação mecânica, de uma analgesia adequada, da estratégia ventilatória protetora, de um protocolo de desmame, da cabeceira elevada, de lavarmos as mãos - todas são intervenções com grande resultado e baixíssimo custo. As recomendações brasileiras de ventilação mecânica, que têm como objetivo principal agregar valor à ventilação mecânica, foram publicadas em duas partes, devido a sua extensão e abrangência, por duas revistas brasileiras: o Jornal Brasileiro de Pneumologia^(^ 1 ^,^ 2 ^)^ e a Revista Brasileira de Medicina Intensiva.^(^ 3 ^,^ 4 ^)^

A insuficiência respiratória é uma doença frequente, e, para os casos potencialmente reversíveis, o suporte ventilatório é salvador de vidas. Extrapolando os dados epidemiológicos dos EUA,^(^ 5 ^)^ de 2,8 pacientes em ventilação mecânica a cada 1.000 pacientes/ano, para a realidade brasileira (população atual segundo o Instituto Brasileiro de Geografia e Estatística de 203.175.000 habitantes),^(^ 6 ^)^ podemos estimar que cerca de 570.000 pacientes necessitem de suporte ventilatório invasivo a cada ano. Simulando um tempo médio de ventilação mecânica de três dias, chega-se a 1.706.670 pacientes/dia em ventilação mecânica, e, com as informações do Censo da Associação de Medicina Intensiva Brasileira de 2011,^(^ 7 ^)^ que calculou haver no Brasil cerca de 25.000 leitos de UTI, podemos estimar que, a cada dia, 19% dos leitos de UTI serão utilizados por pacientes intubados. Ainda pegando emprestado os dados americanos,^(^ 5 ^)^ cuja estimativa do custo da internação hospitalar para um paciente com insuficiência respiratória e com necessidade de ventilação mecânica é de US$ 34.000, podemos extrapolar que o Brasil deve gastar algo em torno de R$ 54,5 bilhões/ano, considerando-se 12% do gasto em saúde^(^ 8 ^)^ e 1,1% do PIB^(^ 9 ^)^ para o tratamento hospitalar de pacientes com insuficiência respiratória aguda ou crônica agudizada.

Os números expostos acima, com a ressalva de serem frutos de um simples exercício epidemiológico, matemático e financeiro, alerta para o enorme impacto da insuficiência respiratória e da ventilação mecânica para a política de saúde em nosso país. Por outro lado, é importante lembrar que o suporte ventilatório é sabidamente um tratamento custo-efetivo para a maior parte dos pacientes. Estudos publicados nos últimos anos mostram valores de US$ 26.000 a US$ 175.000 para *quality-adjusted life-year* (QALY, ano de vida ganho ajustado por qualidade de vida), dependendo da etiologia da insuficiência respiratória, das comorbidades e da idade do paciente.^(^ 10 ^)^ Apesar de arbitrário, é prática atual aceitar como custo-efetivos os tratamentos resultando em US$ 50.000-150.000/QALY.^(^ 11 ^)^

As recomendações publicadas^(^ 1 ^-^ 4 ^)^ ressaltam que os resultados do tratamento de pacientes com insuficiência respiratória aguda melhoraram muito nas últimas décadas, e o mais interessante é que esse avanço veio muito mais do melhor entendimento da fisiopatologia da insuficiência respiratória e da prevenção de complicações associadas à ventilação mecânica do que do desenvolvimento de novas drogas ou tecnologias. Os ventiladores mecânicos, na sua essência básica, mudaram muito pouco nesse período, mas a forma do seu uso mudou completamente, evoluindo de uma estratégia agressiva para a correção da hipoxemia e/ou hipercapnia para uma estratégia focada em ofertar uma ventilação alveolar mínima para garantir as trocas gasosas, poupando os pulmões de lesões adicionais, e, assim, dando o tempo necessário para a sua recuperação. O Brasil teve uma participação decisiva no desenvolvimento desses conceitos modernos de ventilação mecânica, particularmente no entendimento da fisiopatologia da SARA e no pioneirismo em demonstrar as vantagens da estratégia ventilatória protetora.^(^ 12 ^,^ 13 ^)^ Apesar disso, dados recentes mostram que a mortalidade de pacientes em ventilação mecânica em nosso meio ainda é alta quando comparada com a de países desenvolvidos.^(^ 14 ^)^ A dificuldade de acesso a leitos de UTIs, o número e a qualificação insatisfatória de profissionais de saúde destacados para o cuidado de pacientes com insuficiência respiratória, os equipamentos obsoletos e, principalmente, a falta de aderência às melhores práticas de cuidado são fatores que certamente contribuem para esse resultado preocupante.

As recomendações brasileiras de ventilação mecânica 2013^(^ 1 ^-^ 4 ^)^ são uma importante iniciativa. Feitas por profissionais competentes e experientes, elas trazem o estado da arte em ventilação mecânica, apresentado de maneira clara e objetiva e com a preocupação de adequar essas recomendações à forma como praticamos a medicina intensiva em nosso país.

Apesar de reconhecer todo o esforço individual e coletivo dos autores e coordenadores dessa obra, precisamos estar conscientes de que essa é a etapa mais fácil do caminho em busca de um cuidado melhor para os pacientes em ventilação mecânica; o difícil, o grande desafio, não só aqui, mas em todo o mundo, é transformar recomendações e boas intenções em valor para os pacientes.^(^ 15 ^)^ Ressalto que a maioria das recomendações aqui apresentadas não requer novas tecnologias ou maiores recursos financeiros e são, em grande parte, intuitivas e já conhecidas pelos profissionais que trabalham nas UTIs em nosso meio. Temos outro complicador: como implementá-las em um país com as características do Brasil: um país continental, heterogêneo, onde somos criativos mas pouco disciplinados em seguir recomendações, com carência de profissionais qualificados, sem cultura de treinamento e de educação profissional continuada para profissionais da saúde e com pouca mensuração da real qualidade do cuidado oferecido nas instituições de saúde pública ou privadas.

Os autores dessas recomendações^(^ 1 ^-^ 4 ^)^ fizeram a sua parte, e temos mais um excelente guia na direção à ventilação mecânica com mais qualidade, segurança e valor para pacientes com insuficiência respiratória. O conteúdo e o racional estão postos; agora é hora de transformarmos essas evidências e recomendações em prática, e isso só irá acontecer com trabalho, disciplina e envolvimento de cada um de nós. Mãos a obra!
